# Synchronized bilateral percutaneous nephrolithotripsy in a horseshoe kidney

**DOI:** 10.4103/0970-1591.38619

**Published:** 2008

**Authors:** R. Krishna Rao, M. S. Ranganath, T. Krishna Prasad

**Affiliations:** Urology Department, Vikram Hospital and Heart Care, Mysore, Karnataka, India

**Keywords:** Horseshoe kidney, percutaneous nephrolithotripsy, stag horn

## Abstract

Stone management in horseshoe kidneys is challenging: percutaneous nephrolithotripsy is a safe and effective procedure in the treatment of large stones within horseshoe kidneys. Simultaneous bilateral PCNL has been described with a single surgeon operating sequentially on one renal unit following the other under the same anesthetic. We describe synchronized bilateral PCNL by two operating teams in tandem for a patient with bilateral stag horn calculi associated with a horseshoe kidney.

## CASE REPORT

**Figure 1:**
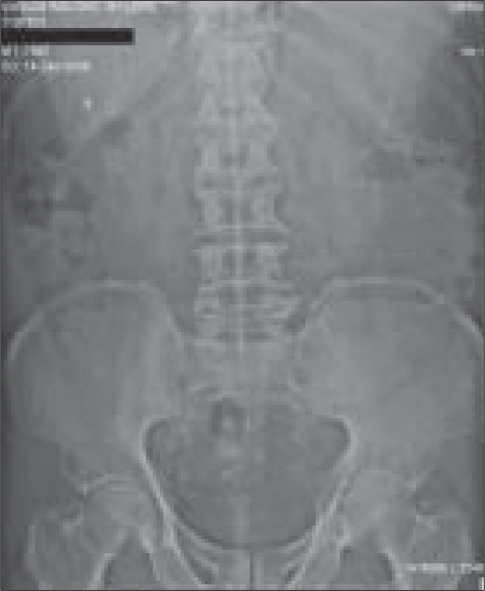
Pre operative KUB showing bilateral staghorn stones

**Figure 2:**
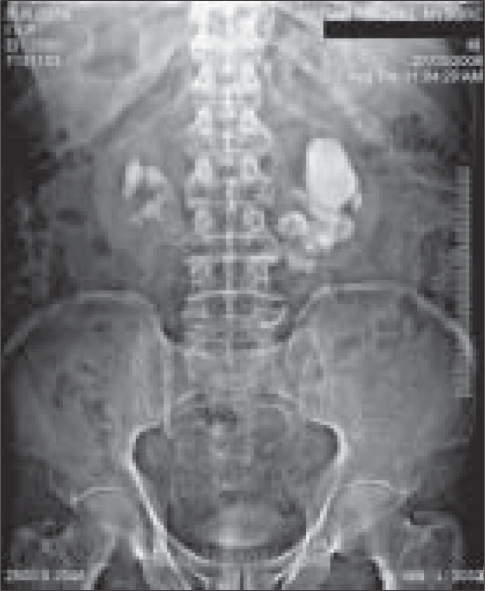
Pre operative IVU showing horseshooe kidney, hydronephrosis and bilateral staghorn stones

**Figure 3:**
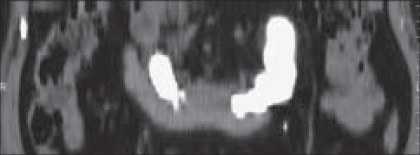
CT coronal confirming horseshoe kidney and bilateral stones

A 73-year-old male presented with left loin pain and hematuria. Ultrasound revealed bilateral stag horn calculi in a horseshoe kidney, which was confirmed by intravenous urogram and spiral CT [Figures 1-3]. Renal function was normal.

Since there was cardiac co-morbidity the decision taken was to limit the number of stages required to clear the stones. Synchronized PCNL to clear the bulk of the stones in one sitting was undertaken in order to achieve this objective.

**Figure 4:**
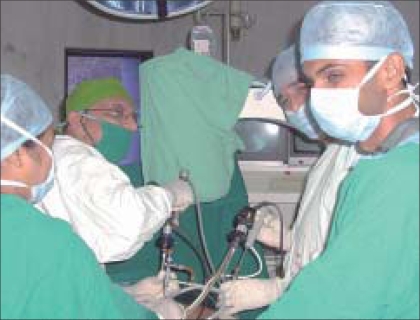
OT ergonomics optimizing utilization of space

Pre-procedure planning included deployment of the C arm which was positioned on the right side of the patient [Figure 4]. After bilateral retrograde ureteric catheter placement, tracks were placed sequentially by one surgeon standing first on the patient's left and then on the right side without shifting the C arm. The slightly cramped position did not prove a problem.

**Figure 5:**
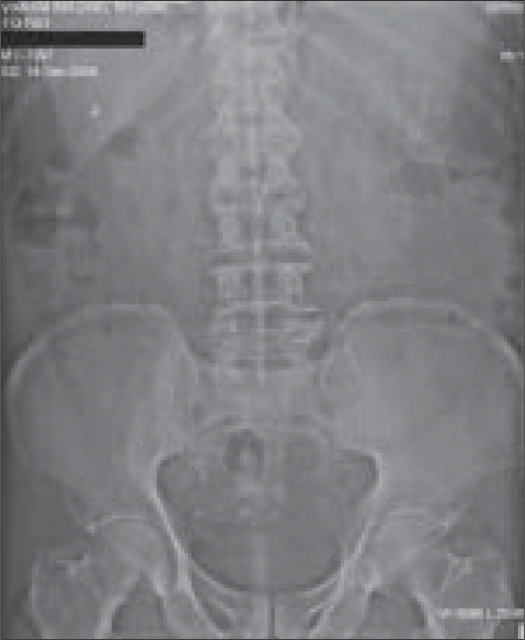
KUB at 4 months no significant residual fragments

The tracks were at an angle of 45 to 90° to each other, resulting in the two surgeons looking in opposite directions at their individual monitors without hindrance. The camera and light cables, irrigation sets and other accessories did not get entangled in this position as both team leaders, being experienced at PCNL, were conscious of the coordination required. An ultrasound lithotripter was used on the left and the Swiss Lithoclast on the right side.

The procedure took 124 min. Only the deeply placed, medially directed, inferior calyx on the left side proved difficult to reach. This was cleared at a brief second look PCNL four days later.

There was no significant bleeding, transfusion was not required and complete clearance was achieved. Postoperative recovery was uneventful. KUB Xray [Figure 4] and ultrasound scan four months later revealed well-functioning kidneys and complete stone clearance. Renal function remained normal throughout.

## DISCUSSION

PCNL has been shown to be the ideal method for stone clearance in horseshoe kidneys.[1] However, it can pose unique problems, particularly if there are large stag horn calculi. We investigated a synchronous bilateral approach with two teams lead by two experienced surgeons. Though there is a mention in the literature of simultaneous bilateral PCNL, what actually is done is a sequential PCNL by one surgeon.[2–5]

There are several advantages to the synchronized approach. Firstly, with two teams, operating time to clear both kidneys is reduced. The need for multiple staged procedures is thus obviated. This is of significant importance in an older patient such as ours with significant co-morbidities. Simultaneous sequential PCNL requires the successful completion of one side before proceeding to the other. There is therefore no certainty that the second side can indeed be performed under the same anesthetic, the decision to proceed being taken at the end of the first procedure.[2]

Operating and anesthetic time are greater with sequential PCNL than when both sides are done synchronously. Additional time is required between the two sides to reposition and redeploy the equipment.

The theoretical possibility is of simultaneous major complication occurring in both kidneys at the same time when two teams operate. These can however be tackled using basic principles, e.g. tamponade for bleeding, nephrostomy tube placement and termination of the procedure if indicated.

Synchronous PCNL requires two experienced surgeons working together, assistants and nursing staff familiar with the technique and two complete sets of instruments including telescopes, cameras, monitors, energy sources and grasping instruments. A solitary C arm is adequate as the imaging modality, but necessitates preliminary planning of track placement which has to be done sequentially.

Two teams operating in tandem have the advantage of reducing the number of sittings required to clear these difficult calculi. Overall anesthetic and operating time are also reduced with the potential of minimizing blood loss, transfusion requirements and morbidity.

While our experience is obviously limited, the procedure is feasible. Limitations are posed by the availability of equipment, accessories and the experience of the operating teams.

## CONCLUSION

This first description of synchronous PCNL in a horseshoe kidney by two operating teams indicates that the procedure is feasible, safe and worthy of further evaluation.
